# Gross intermittent hematuria after laparoscopic donor nephrectomy

**DOI:** 10.4103/0972-9941.40993

**Published:** 2008

**Authors:** G Gaurav, K Santosh, A Samiran, G Ganesh

**Affiliations:** Department of Urology, Christian Medical College, Vellore, Tamil Nadu, India

**Keywords:** Laparoscopic, donor nephrectomy, complications

## Abstract

Laparoscopic donor nephrectomy is a routine practice but still requires an intense level of attention to prevent complications. We report a rare case of gross hematuria in postoperative period after an uneventful laparoscopic donor nephrectomy.

## INTRODUCTION

Laparoscopic donor nephrectomy (LDN) has gained wide acceptance having a substantial impact on donor operation by providing a less invasive approach and without jeopardizing procurement of a high quality renal allograft.[1] The lower incidence of serious peri-operative morbidity in LDN is well documented. We report a case of gross hematuria from ureteral stump occurring during postoperative period after an uneventful LDN. Diagnostic and management issues are discussed.

## CASE REPORT

**Figure 1:**
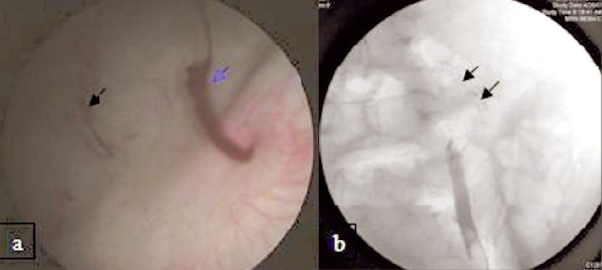
(a) Cystoscopic image showing small tubular clot (black arrow) in the bladder and jet of blood from left ureteric orifice (blue arrow). (b) Retrograde study showing spillage of contrast out of the ureteric stump (arrow), amount of spillage depends on pressure with which contrast was injected

**Figure 2:**
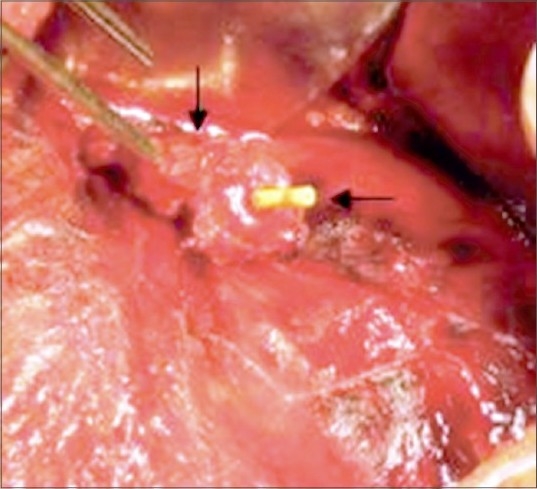
Per-operative photograph showing ureteric catheter (horizontal arrow) through the ureteral stump (vertical arrow)

A healthy 19-year-old male underwent an uneventful left trans-peritoneal LDN with extraction of kidney from small extension of 10-mm laparoscopic port in left iliac fossa. As is standard practice, the gonadal vein was clipped and transected laparoscopically and ureter alone was ligated with number 1 chromic catgut at the time of graft retrieval. Urine was clear postoperatively and the urethral catheter was removed on first postoperative day. After 24 h, he developed sudden gross intermittent hematuria and had clot retention. A hematuria catheter was placed; bladder wash was given followed by bladder irrigation. Serum creatinine was normal and the patient was hemodynamically stable. He had a fall of hematocrit from 37 to 26%. As the hematuria was recurrent and there was a significant fall in hematocrit, blood was transfused and he was taken for exploration. Cystoscopy revealed a small clot in the bladder and intermittent jet of blood from left ureteric orifice [Figure 1a]. Retrograde study showed spillage of contrast out of the ureteric stump [Figure 1b] and ureteroscopy showed blood clot in the ureter without any identifiable bleeding source. He underwent exploration through the same incision from where the kidney was procured. The catgut ligature over the ureter had slipped and the ureteric catheter, which was placed after ureteroscopic examination, was visible through the ureteral stump [Figure 2] with surrounding small hematoma. No active intraabdominal bleeding could be identified. The ureteric stump was mobilized and doubly ligated with 2-0 vicryl.

Postoperative period was uneventful and Foley catheter was removed on the second postoperative day. He was discharged 48 h after catheter removal. On follow-up, he was absolutely asymptomatic and hematocrit was stable with clear urine output.

## DISCUSSION

Hematuria through the ureteral stump after LDN is extremely rare.[2] In one of the largest series of laparoscopic donor nephrectomy the authors have reported no major or minor complications associated with the ureteral stump in the donor.[3] In healthy donors who present with hematuria after LDN a high index of suspicion for bleeding through the ureteral stump should be considered. Live kidney donors are healthy individuals who have had an extensive evaluation including a contrast computed tomography and are unlikely to harbor a preexisting pathology to contribute for the hematuria. Imaging studies may or may not demonstrate[4] conclusive findings thus delaying the definitive treatment. In any transplant program, donor safety is of utmost importance, requiring aggressive management of the complications.
